# Land Use and Stream Nitrogen Concentrations in Agricultural Watersheds Along the Central Coast of California

**DOI:** 10.1100/tsw.2001.315

**Published:** 2001-11-22

**Authors:** Marc Los Huertos, Lowell E. Gentry, Carol Shennan

**Affiliations:** Department of Environmental Studies, University of California, Santa Cruz 95064, USA

**Keywords:** marine sanctuary, water quality, nitrate, load, surface water, National Estuarine Research Reserve

## Abstract

In coastal California nitrogen (N) in runoff from urban and agricultural land is suspected to impair surface water quality of creeks and rivers that discharge into the Monterey Bay Sanctuary. However, quantitative data on the impacts of land use activities on water quality are largely limited to unpublished reports and do not estimate N loading. We report on spatial and temporal patterns of N concentrations for several coastal creeks and rivers in central California. During the 2001 water year, we estimated that the Pajaro River at Chittenden exported 302.4 Mg of total N. Nitrate-N concentrations were typically <1 mg N l_–1_ in grazing lands, oak woodlands, and forests, but increased to a range of 1 to 20 mg N l_–1_ as surface waters passed through agricultural lands. Very high concentrations of nitrate (in excess of 80 mg N l_–1_) were found in selected agricultural ditches that received drainage from tiles (buried perforated pipes). Nitrate concentrations in these ditches remained high throughout the winter and spring, indicating nitrate was not being flushed out of the soil profile. We believe unused N fertilizer has accumulated in the shallow groundwater through many cropping cycles. Results are being used to organize landowners, resource managers, and growers to develop voluntary monitoring and water quality protection plans.

## INTRODUCTION

The Monterey Bay National Marine Sanctuary is the largest marine protected area in the U.S., consisting of more than 8000 $km^2$ along the central coast of California. The Sanctuary also includes Elkhorn Slough, a National Estuarine Research Reserve and one of the largest remaining tidal wetlands in California[1]. In general, the marine water in the Monterey Bay is well mixed and deep ocean upwelling is likely to be the primary source of nitrogen (N). However, estuarine and freshwater habitats that experience muted tidal action have elevated N concentrations[2,3,4].

The three main watersheds that drain into the coastal habitats of the Monterey Bay Sanctuary are the Pajaro River (3394 $km^2$), Elkhorn Slough (203 $km^2$), and Salinas River (10,600 $km^2$). Within these three watersheds, agriculture and livestock production accounts for approximately 75% of the total land area[5]. Other land uses include managed timber, protected open space and parks, and urban and suburban development. With a mild coastal Mediterranean climate and fertile soils, the region produces 11% or $2.5 billion worth of total agricultural receipts in the state[6].

The regional water quality agency has determined that major waterways draining into the sanctuary do not meet standards for their designated beneficial uses. The cause of impairment includes elevated nutrients, nuisance algae, sediment load, and pesticide contamination[2,3,4,7,8]. The role of land use on N loading and the subsequent effect of N loading on water quality are largely unknown. Most water quality data for the region are in unpublished reports, and except for two studies[2,3,4], monitoring data have not explicitly linked land use with water quality. Finally, no loading estimates have been made to date, which will be needed to develop TMDL (Total Maximum Daily Load) guidelines. Building on previous work, we developed a monitoring program to link land use with nutrient concentrations and loading in the Pajaro River and Elkhorn Slough watersheds. We sampled water in rivers, creeks, and agricultural drainage ditches to characterize the relationship between land use and N concentrations. We used discharge measurements to calculate N loading in the Pajaro River and Elkhorn Slough watersheds. In this paper we present results from the 2001 water year that will be used to coordinate landowners, resource managers, and growers to develop voluntary monitoring and water quality protection plans.

## PROCEDURES

We collected water samples at 35 sites located throughout the Pajaro River and Elkhorn Slough watersheds on a biweekly basis during the 2001 water year (October 1, 2000 to September 30, 2001). These sites were selected to target water sources of the Monterey Bay that have important consequences for wetland and near-shore habitat. In addition to biweekly sampling, several locations were sampled more intensively to capture storm event variability. For brevity we report data for eight stream sites and three agricultural drainage ditches that exemplify the overall patterns observed (Fig. 1).

We sampled the Pajaro River at Chittenden (site 1) and at Main Street in the city of Watsonville, CA (population of 47,000) (site 2). Above Chittenden, land use is a mix of grazing land, intensive row crop agriculture, urban and suburban development, oak woodland, conifer forests, and irrigated hay fields. Downstream of Chittenden, the Pajaro River passes through grazing land, row crop agriculture, and orchards until entering the Pajaro Valley, where row crop agriculture is the predominant land use. The Pajaro River then flows through the city of Watsonville and thereafter through a large, continuous area of vegetable and strawberry production before emptying into the Monterey Bay. Sewage effluent from the city of Watsonville is treated and then discharged offshore in the Monterey Bay, bypassing the Pajaro River.

Corralitos Creek is the largest tributary entering the Pajaro River between Chittenden and the Monterey Bay. We sampled Corralitos Creek at two locations, Las Colinas Road (site 3) and Green Valley Road in Watsonville (site 4). The watershed above Las Colinas Road is dominated by a redwood forest. Land use below Las Colinas Road is characterized by orchards, row crops, and suburban and urban development.

West of Watsonville there is a large wetland complex. Within this region, we sampled a channelized wetland called Watsonville Slough at Errington Road (site 5) and San Andreas Road (site 6). Above Errington Road the slough begins as urban runoff from Watsonville. Below Errington Road the slough passes through strawberry and vegetable crops and merges with two other sloughs above San Andreas Road. Farther downstream the slough enters a tidal wetland and mixes with the Pajaro River and Monterey Bay waters.

Along both sides of the Pajaro River below the city of Watsonville are fields in row crop production. Most of these fields are drained by a series of subterranean perforated pipes (tiles) installed 1 to 2 m below the soil surface. Without tile drainage these soils would not be suitable for year-round cultivation due to a perched water table. We sampled three agricultural drainage ditches (locations not shown) that frequently received water from tile drains.

In the Elkhorn Slough Watershed we sampled Carneros Creek (which is the primary freshwater source for the Elkhorn Slough) at Dunbarton Road (site 7) and downstream at San Miguel Canyon Road (site 8). The upper part of the watershed is a mix of grazing land and oak woodland. The lower part of the watershed consists of rural ranchettes, strawberry and cut-flower production fields, and horse stables.

Water samples were analyzed in the field for temperature, dissolved oxygen, conductivity, and pH with a Hydrolab Surveyor 3 multiprobe sensor (Austin, TX). Water samples were filtered (Pall type A/E glass fiber) within 24 h of collection and then frozen. Samples were analyzed for nitrate-N (nitrate-N + nitrite-N) and ammonium-N, within 48 h using a Lachat 8000 series autoanalyzer (Zellweger Analytics, Milwaukee, WI)[9]. During high flow events additional samples were collected to determine total N. We estimated organic N [organic N = (total N) - (nitrate-N + ammonium-N)] by digesting unfiltered aliquots with persulfate and analyzed for total N using the Lachat autoanalyzer[9].

Rainfall measurements in the Pajaro River watershed were determined by using data from the California Irrigation Management Information Systems (CIMIS) stations #111 (Watsonville, CA) and #126 (Hollister, CA)[10]. We determined rainfall in the Elkhorn Slough watershed near San Miguel Canyon Road using a Campbell Scientific tipping bucket and CR10 datalogger (Logan, UT).

The U.S. Geological Survey determined flow rates for the Pajaro River at Chittenden (Lat. 36°54′01″N, Long. 121°35′48″W) and for Corralitos Creek at Green Valley Road (Lat. 36°56′2″N, Long. 121°46′10″W). The total watershed drainage area of the Pajaro River at Chittenden and of Corralitos Creek at Green Valley Road was 3073 and 72 $km^2$, respectively. We determined flow of Carneros Creek at San Miguel Canyon Road (Lat. 36°51′29″N, Long. 121°41′41″W) using a Unidata Starflow Model 6526B (Willetton, Australia). The watershed drainage area at San Miguel Canyon Road was 85 $km^2$. Also, at this site we used an ISCO 2900 (Lincoln, NE) to automatically collect water samples on a sequential time regiment. By employing an automatic water sampler we were able to characterize every flow event during the 2001 water year, and a total of 185 samples were collected and analyzed. For the three gauged sites, we estimated N loading during the 2001 water year by interpolating N concentrations between sampling events and multiplying by average flow rates. Using previously collected data, we calculated a nitrate-N load at Chittenden for the 1998 water year[2].

## RESULTS AND DISCUSSION

### Pajaro River Watershed

#### *Pajaro River*

During the 2001 water year, 334 mm of precipitation occurred at Hollister (CIMIS station #126) compared to the 50-year average of 343 mm. Although near the annual average, precipitation during the wettest months (November to March) was below aver-

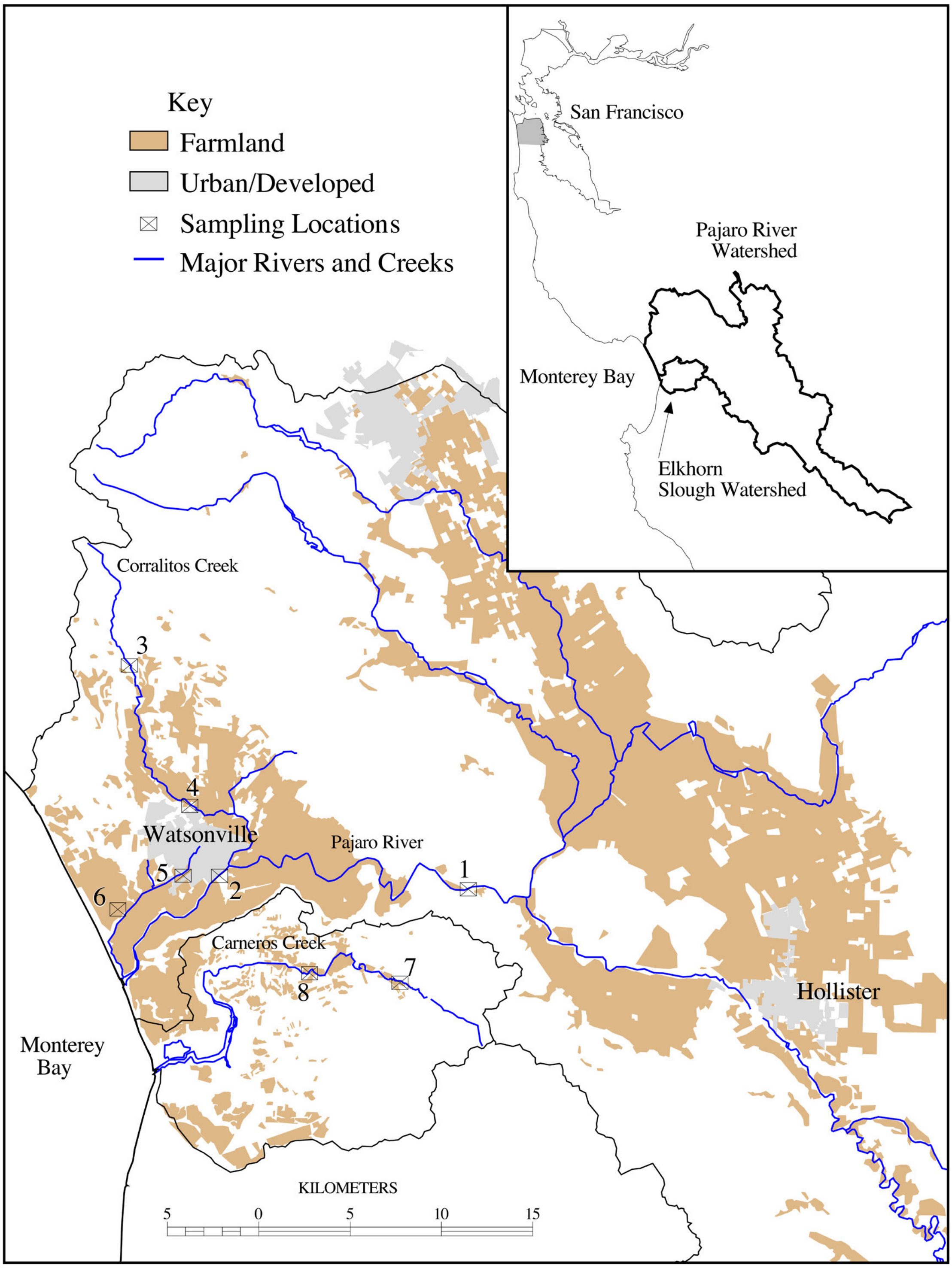

**FIGURE 1**. Watershed boundaries, agricultural land use, and sampling locations: 1. Chittenden, Pajaro River; 2. Main Street, Pajaro River; 3. Las Colinas, Corralitos Creek; 4. Green Valley Road, Corralitos Creek; 5. Errington Road, Watsonville Slough; 6. San Andreas Road, Watsonville Slough; 7. Dunbarton Road, Carneros Creek; 8. San Miguel Canyon, Carneros Creek.

age, which produced limited runoff compared with other years in the past decade (Fig. 2). Total flow for the Pajaro River at Chittenden during the 2001 water year was 52.3 million $m^3$ and nitrate-N concentrations ranged from 1.7 to 20.4 mg $l^{-1}$ with a flow-weighted mean of 5.8 mg $l^{-1}$ (Fig. 3). Although nitrate-N concentrations were diluted by high flows during the winter months, N loading was highest at this time (data not shown). Organic-N was negligible except during high flows with a range of <0.02 to 0.89 mg $l^{-1}$. Ammonium-N concentrations were low throughout the year with a range of 0.01 to 0.08 mg $l^{-1}$. We esti-

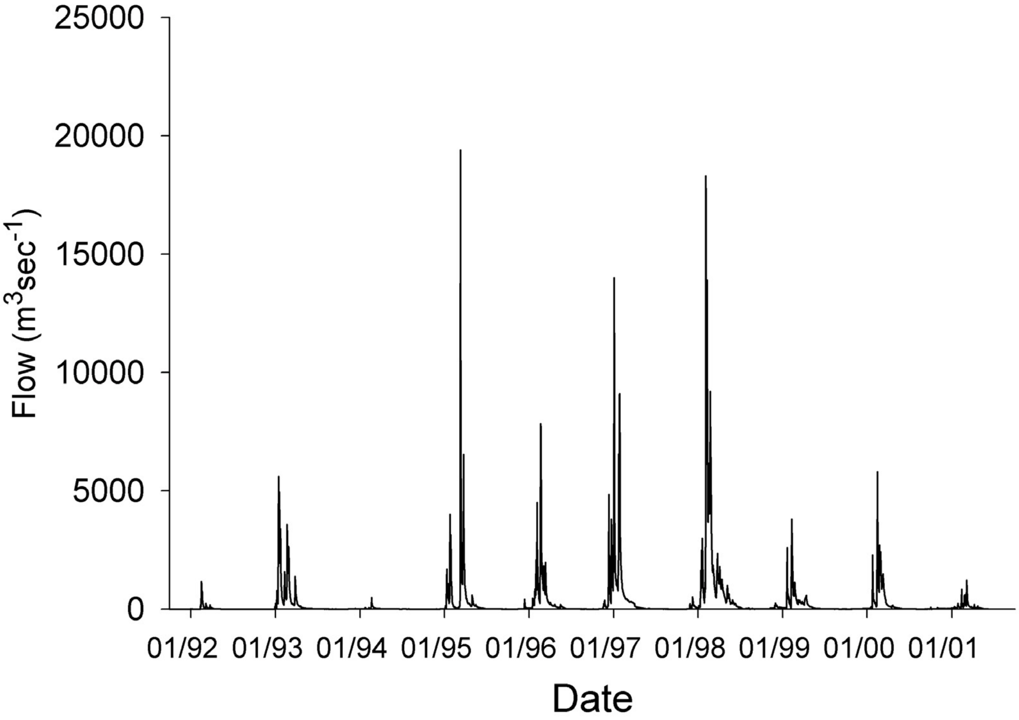

**FIGURE 2**. Discharge for the Pajaro River at Chittenden, water years 1992–2001.

mated the total annual N load in the Pajaro River at Chittenden to be 302.4 Mg of which 96.5% was nitrate-N, 2.8% organic-N, and 0.7% ammonium-N.

In contrast, 1998 was the wettest water year in the past decade, and Hollister received 719 mm of precipitation, which produced a total flow at Chittenden of 765 million $m^3$ (Fig. 2). We calculated a nitrate-N load of 1374.7 Mg at Chittenden during the 1998 water year, compared with 292.0 Mg for the 2001 water year. Although flow during the 2001 water year was only 7% of the 1998 water year, the nitrate-N load was 21%. Based on the entire watershed area above Chittenden, annual river nitrate-N loads represent the loss of 4.47 and 0.95 kg N $ha^{-1}$ from terrestrial sources for the 1998 and 2001 water years, respectively.

Downstream of Chittenden, the Pajaro River passes through agricultural lands and mixes with water from Corralitos Creek and urban runoff from the City of Watsonville before arriving at Main Street. The river reach between Chittenden and Main Street has been shown to loose a substantial amount of flow, especially during the summer and fall (Jonathan Lear, 2001, personal communications). Although flow at the Chittenden and Main Street sites occurred without interruption, at a sampling site approximately midway between Chittenden and Main Street, river flow ceased in mid July 2001 and did not resume prior to the end of the water year. Therefore, after mid-July we could not ascertain the definitive source of flow at Main Street.

During our study, the Pajaro River exhibited continuous surface flow between Chittenden and Main Street from October 1, 2000 to July 18, 2001. During this time, nitrate-N concentrations at Main Street were usually lower than at Chittenden with a range of 2.0 to 12.3 mg $l^{-1}$. Although the Pajaro River passes through 5 km of intensively cropped lands above Watsonville, nitrate-N concentration decreased an average of 22%. This decline was probably due to dilution by the inflow of lower N water from Corralitos Creek and urban runoff, although we cannot exclude the potential role of instream N processes in reducing nitrate concentrations[11]. After surface flow became discontinuous between Chittenden and Main Street, nitrate concentrations at Main Street remained in a small range of 8.1 to 9.7 mg $l^{-1}$ (Fig. 3). Ammonium-N concentrations were low throughout the year at Main Street and ranged from 0.01 to 0.08 mg $l^{-1}$, similar to Chittenden.

Nitrate-N concentrations in the Pajaro River at Chittenden exceeded the U.S. drinking water standards of 10 mg $l^{-1}$ in May, peaked in August, and remained at these high levels through the end of the water year (Fig. 3). These levels are likely to cause eutrophication, but there are no state standards to assess this potential. During low flow periods, high nitrate concentrations add little to annual river load, but may adversely impact groundwater quality[12].

## Corralitos Creek

During the 2001 water year 473 mm of rainfall occurred at Green Valley Road (located at the base of the Corralitos watershed) compared with the 50-year average of 574 mm. The nitrate-N concentration at Las Colinas Road, the upstream site on Corralitos Creek, was usually <0.1 mg $l^{-1}$ with a high of 0.24 mg $l^{-1}$ (Fig. 3). Ammonium-N ranged from <0.01 to 0.07 mg $l^{-1}$. The data indicate N loss in runoff from forested lands above Las Colinas Road was small. Below Las Colinas Road, Corralitos Creek passes

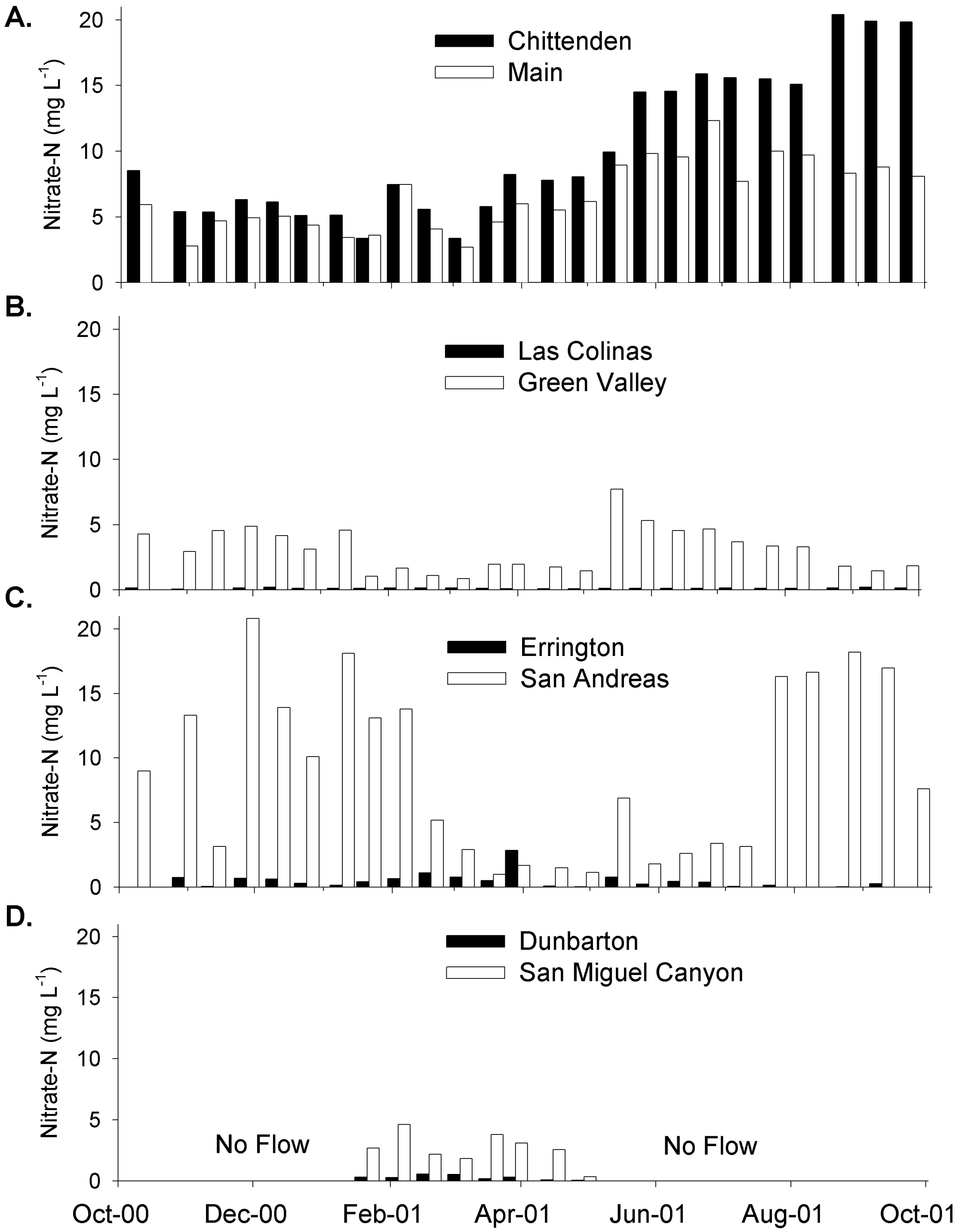

**FIGURE 3**. Nitrate-N concentration in biweekly grab samples, comparing upstream (filled bars) and downstream (empty bars) locations in four water reaches. A. Pajaro River; B. Corralitos Creek; C. Watsonville Slough; D. Carneros Creek.

through suburban developments, orchards, and vegetable production, which contributed to elevated nitrate-N concentrations. Although flow stopped in May at sampling locations between Las Colinas and Green Valley, flow at Green Valley occurred throughout the water year. Nitrate-N concentration at Green Valley Road ranged from 0.6 to 7.7 mg $l^{-1}$ with a flow weighted mean of 1.2 mg $l^{-1}$. Organic-N was negligible except during high flows with a range of <0.02 to 0.36 mg $l^{-1}$ and ammonium-N ranged from 0.02 to 0.08 mg $l^{-1}$. Annual total N load in Corralitos Creek at Green Valley Road was 7.9 Mg (86.6% nitrate-N, 10.2% organic-N, and 3.2% ammonium-N) representing a terrestrial loss of 0.11 kg N $ha^{-1}$.

**TABLE 1**
**Nitrate-N Concentrations (mg $l^{-1}$) in Selected Surface Water Drainage Ditches**

| | 2/13/01 | 3/13/01 | 4/10/01 | 5/22/01 | 6/5/01 | 7/2/01 | 8/01/01 | 9/4/01 |
|---|---|---|---|---|---|---|---|---|
| Drainage Ditch 1 | 21.5 | 19.9 | 23.0 | 24.6 | 23.3 | 24.2 | 21.7 | 22.8 |
| Drainage Ditch 2 | 88.7 | 89.5 | 76.5 | 79.0 | 85.5 | 76.0 | 63.7 | 63.5 |
| Drainage Ditch 3 | 81.2 | 80.7 | 63.7 | 75.1 | 82.3 | 79.2 | 66.3 | 66.1 |

### *Watsonville Slough*

By sampling at Errington Road, we determined that runoff from Watsonville contained low nitrate-N with a range of <0.02 to 2.8 mg $l^{-1}$. Ammonium-N concentrations were usually low, although we found concentrations above 0.1 mg $l^{-1}$ on five sampling dates. Overall, ammonium-N concentrations ranged from 0.02 to 0.38 mg $l^{-1}$ with a mean of 0.11 mg $l^{-1}$. Just 4 km downstream at San Andreas Road, nitrate-N concentrations were higher, often above 10 mg $l^{-1}$ (Fig. 3). Conversely, ammonium-N concentrations at San Andreas Road were lower and ranged from 0.01 to 0.23 mg $l^{-1}$ with a mean of 0.07 mg $l^{-1}$. Land adjacent to the slough consisted of intensive row crop agriculture with no suburban development; therefore, row crop agriculture was the dominant source of nitrate in Watsonville Slough above San Andreas Road.

The nitrate concentration in Watsonville Slough at San Andreas Road decreased in February and remained low through April (Fig. 3). We believe this decline was primarily due to dilution from storm water runoff. In general, nitrate concentrations at San Andreas Road during the dry months were variable (Fig. 3). After inflows from adjacent sloughs decreased, the water of the Watsonville Slough became stagnant. We found dissolved oxygen concentrations decreased to <5 mg $l^{-1}$ in June and remained low for the remainder of the water year, indicating development of eutrophic conditions and suggesting that instream processing may be an important factor influencing N concentrations throughout the summer months[11].

### *Agricultural Drainage Ditches*

We found high nitrate concentrations in the three agricultural drainage ditches sampled in the Pajaro River Valley (Table 1). These ditches frequently received discharge from tile drainage and exhibited continuous flow throughout the year. We believe that tile discharge is a substantial portion of ditch flow, as well as a major source of nitrate. From early February through September, nitrate concentrations of each ditch were relatively constant over time and did not show dilution following rainfall. Ditches 1, 2, and 3 had average nitrate-N concentrations of 22.6, 77.8, and 74.3 mg $l^{-1}$, respectively (Table 1). These are among the highest nitrate-N concentrations reported for any water type in this region[3]. Ammonium-N concentrations in these ditches ranged from 0.01 to 0.08 mg $l^{-1}$, indicative of baseflow in tiles[13]. These results suggest this area of the lower Pajaro River Valley contains a shallow water table that is N saturated. Based on the locations sampled to date, this water table extends at least several square kilometers.

Fields in conventional row crop agriculture typically receive high N application rates, often twice the amount removed by harvest, leaving a substantial amount of N available for leaching[13,14]. We hypothesize that unused N fertilizer in the Pajaro River valley has accumulated in the shallow groundwater through many cropping cycles. Therefore, concerned growers who respond by limiting N inputs may not see short-term water quality improvements in these drainage ditches. Continued monitoring will establish background information needed to evaluate the impact of any future cultural changes.

## Elkhorn Slough Watershed

### *Carneros Creek*

The Elkhorn Slough watershed received 450 mm of precipitation during the 2001 water year. Carneros Creek was ephemeral and flowed between January 9 and April 24, 2001 (Fig. 4). Nitrate concentrations at Dunbarton Road, the upstream site, were always lower than at San Miguel Canyon Road as indicated by the biweekly sampling data (Fig. 3). Similar to the headwaters of Corralitos Creek, nitrate loss from grazing land and oak woodlands above Dunbarton Road was small. However, the combination of agricultural activities and low-density housing lower in the watershed contributed to elevated nitrate concentrations downstream in Carneros Creek.

We estimated total flow at San Miguel Canyon Road during the 2001 water year to be 0.98 million $m^3$. Using the ISCO water sampler we found nitrate-N concentrations at this site ranged from 0.7 to 17.1 mg N $l^{-1}$ with a flow weighted mean of 1.7 mg $l^{-1}$. Organic N concentrations were the highest measured at any site in this study and ranged from <0.02 to 1.38 mg $l^{-1}$, while ammonium-N ranged from 0.03 to 2.30 mg $l^{-1}$. The total N load was 2.7 Mg of which only 70% was nitrate-N. Organic-N contributed 23% of the total N load which reflected a relatively high sediment load compared with the other watersheds (data not shown)[8]. On an area basis, the total N load in Carneros Creek represented a terrestrial loss of 0.03 kg N $ha^{-1}$.

After each storm, nitrate concentration increased in sequential ISCO samples. We speculate that nitrate concentrations increased as baseflow became a higher percentage of the flow compared to surface runoff. These data emphasize the importance of surface-groundwater interactions in determining stream nitrate concentrations.

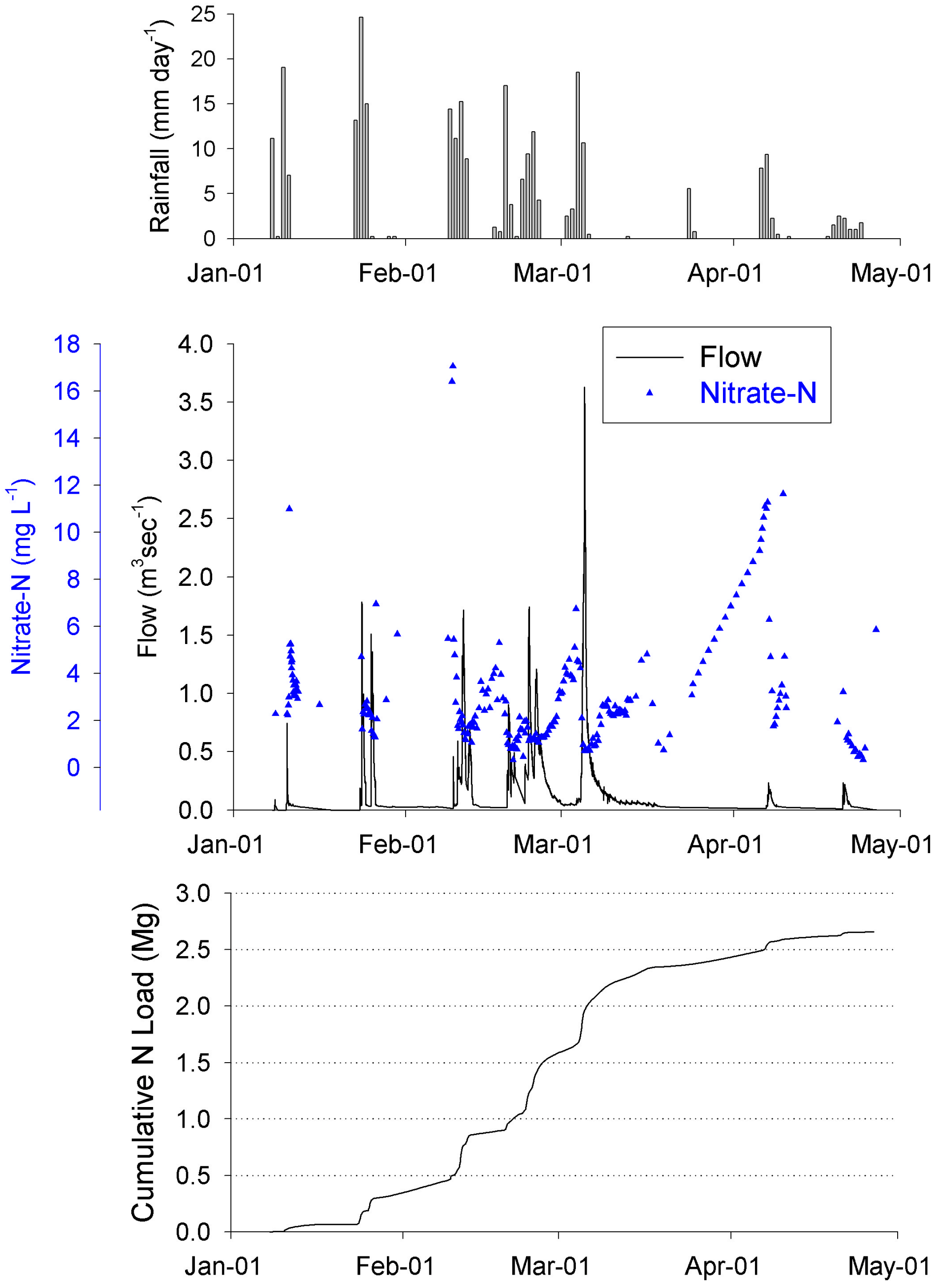

**FIGURE 4**. Top panel: daily rainfall totals in Elkhorn Slough watershed; middle panel: water flow and nitrate-N concentrations at San Miguel Canyon, Carneros Creek; bottom panel: cumulative nitrate-N load at San Miguel Canyon, Carneros Creek, 2001 water year.

## CONCLUSION

In both the Pajaro and Elkhorn Slough watersheds, nitrate concentrations were low in surface water passing through forested and grazing lands, and elevated where intensive agriculture was present. For the Pajaro River at Chittenden and Carneros Creek at San Miguel Canyon Road, we estimated that 302.4 and 2.7 Mg of N were exported during the 2001 water year, respectively. The majority of the N loss was in the form of nitrate, with organic N increasing in importance during high flow conditions. Agricultural ditches receiving tile effluent consistently contained the highest nitrate-N concentrations and did not show dilution effects. We speculate that the shallow, perched water table in the lower Pajaro River valley has accumulated nitrate-N derived from past and present fertilizer applications and remediation may be a long-term process. With the cooperation of resource agencies and grower associations, we will use these results to improve on-farm water quality monitoring, evaluate how practices influence water quality, and help develop water quality protection plans. Future research will investigate possible links between groundwater recharge below Chittenden and the quality of shallow groundwater in the lower Pajaro River Valley.

| | | |
|---|---|---|
| Received: | July | 23, 2001 |
| Revised: | October | 10, 2001 |
| Accepted: | October | 15, 2001 |
| Published: | November | 22, 2001 |
